# Impact of a multifaceted intervention including a smart reminder system for intraoperative antibiotic re-dosing on surgical site infections in a Chinese tertiary care hospital

**DOI:** 10.3389/fpubh.2025.1674811

**Published:** 2025-10-10

**Authors:** Cuiqiong Fan, Guanwen Lin, Huiwen Zhao, Zhenyao Zhao, Baohong Liu, Tian Wang, Ya Zou, Lushi Huang, Zihuan Li

**Affiliations:** ^1^Department of Infection Prevention and Control, The Affiliated Guangdong Second Provincial General Hospital of Jinan University, Guangzhou, China; ^2^Department of Preventive Healthcare and Infection Control, Shenzhen People's Hospital, The First Affiliated Hospital, Southern University of Science and Technology, Shenzhen, China; ^3^Department of Preventive Healthcare and Infection Control, Shenzhen People's Hospital, The Second Clinical Medical College, Jinan University, Shenzhen, China; ^4^Faculty of Humanities and Social Sciences, Hong Kong Metropolitan University, Hong Kong, Hong Kong SAR, China; ^5^Department of Infection Prevention and Control, Guangdong Provincial Social Welfare Service Center, Guangdong Jiangnan Hospital, Guangzhou, China

**Keywords:** smart reminder system, intraoperative antibiotic re-dosing, surgical site infections, healthcare-associated infections, prophylactic antibiotic administration

## Abstract

**Background:**

Surgical site infections (SSIs) are among the most common healthcare-associated infections worldwide. This study evaluated the effectiveness of a multifaceted intervention, which included a smart reminder system for prophylactic intraoperative antibiotic re-dosing in the Anesthesia Information Management Systems, modifications to the preoperative application form, and enhanced review of medical orders, on improving prophylactic intraoperative antibiotic re-dosing and reducing SSIs.

**Methods:**

A retrospective study on the epidemiology of healthcare-associated SSIs was conducted to compare outcomes before and after the implementation of the bundled intervention, with a focus on compliance with intraoperative antibiotic re-dosing and the rate of SSIs.

**Results:**

The proportion of prophylactic antibiotic administration before surgery significantly increased after the intervention (*p* = 0.005). The timing of prophylactic antibiotic administration before surgery was shorter after the intervention, with a median (IQR) of 0.8 (0.6–1.0) h, compared to the before intervention period (*p* < 0.001). The proportion of intraoperative additional antibiotic administration also increased significantly (*p* = 0.003). Furthermore, the rate of SSIs decreased significantly after the intervention (*p* = 0.038).

**Conclusions:**

The multifaceted intervention, comprising a smart reminder system for prophylactic intraoperative antibiotic re-dosing in the Anesthesia Information Management Systems, preoperative application form modifications, and enhanced medical order review, effectively improved intraoperative antibiotic re-dosing compliance and reduced surgical site infection rates.

## Introduction

Surgical site infections (SSIs) are among the most common healthcare-associated infections worldwide, significantly contributing to prolonged hospital stays, increased morbidity and mortality rates, and higher healthcare costs following surgical procedures (1–4). Studies have shown that prolonged operative time can increase the likelihood of developing SSIs across a wide range of surgical procedures (5, 6). The re-dosing of prophylactic antibiotics during lengthy surgeries in patients who underwent surgery lasting ≥3 h is significantly associated with favorable outcomes in terms of the risk of SSIs (7). Despite ongoing efforts to train and emphasize the importance of intraoperative antibiotic re-dosing, particularly for prolonged surgeries (lasting 3 h or more), physicians may fail to promptly assess or determine the need for additional antibiotics due to various factors, such as intraoperative distractions or insufficient smart reminder support. Anesthesia Information Management Systems (AIMS) integrate hospital information systems and perioperative medical devices to digitally record and manage the entire perioperative process. Their use streamlines documentation, improves patient care quality, and supports clinical, research, and administrative functions (8, 9). Thus, this study primarily aimed to retrospectively evaluate the effectiveness of a smart reminder system for prophylactic intraoperative antibiotic re-dosing by comparing re-dosing rates before and after the intervention and assessing its impact on the incidence of SSIs. Through a series of interventions, particularly the integration of information technology and intelligent modules, we aim to contribute to the growing body of evidence on effective strategies to reduce SSIs and enhance patient safety in healthcare settings.

## Materials and methods

### Setting

**Figure 1:**
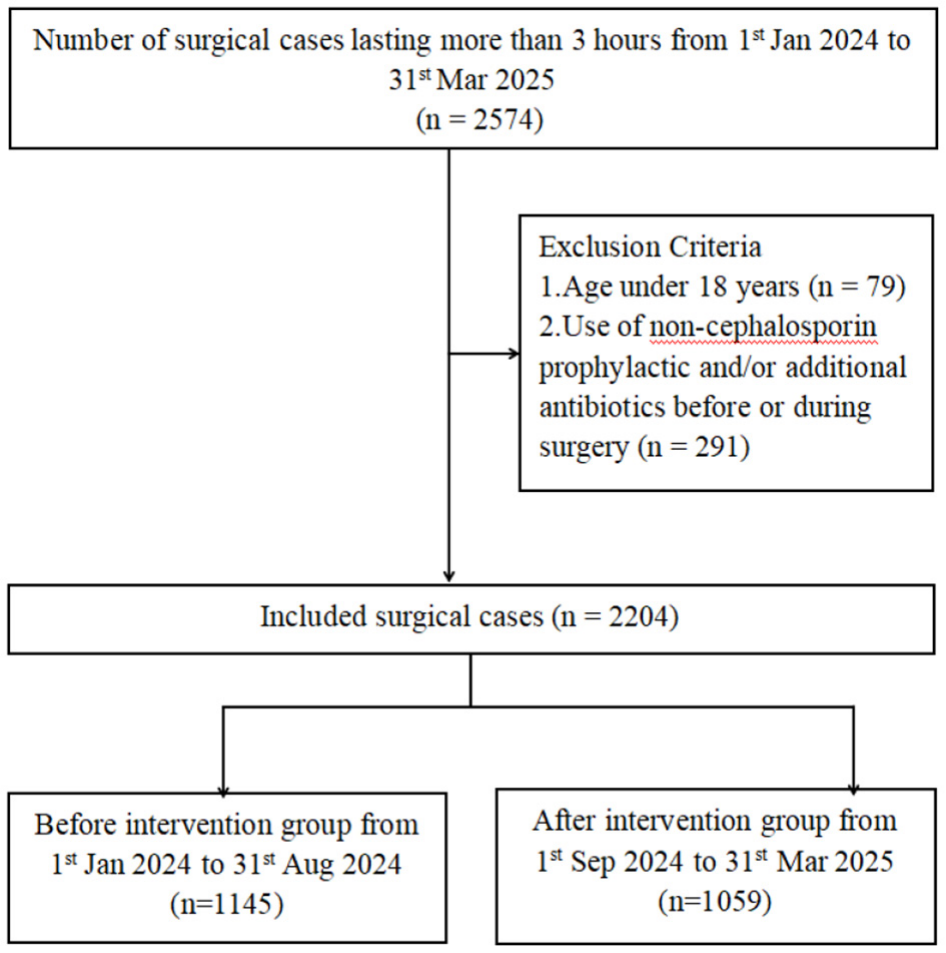
The schematic representation of the study profile.

This is a retrospective study on the epidemiology of healthcare-associated surgical site infections in the Affiliated Guangdong Second Provincial General Hospital of Jinan University, a university-affiliated tertiary hospital located in Guangzhou, Guangdong Province, China, with a bed capacity of approximately 1,730. The study period is divided into two parts, where period 1 (from 1st Jan 2024 to 31st Aug 2024) is the baseline and period 2 (from 1st Sep 2024 to 31st Mar 2025) is the intervention, with the enhancement in the infection control measures (Figure 1).

### Study population and inclusion criteria

Patients who underwent surgery lasting more than 3 h from 1st January 2024 to 31st March 2025, were retrospectively included. Inclusion criteria were age ≥18 years, surgery duration >3 h, and availability of complete medical records, including age, sex, body mass index (BMI), body surface area (BSA), pre-existing comorbidities, hospital resource utilization, surgical characteristics, antibiotic prophylaxis, and other relevant clinical variables. Exclusion criteria included age <18 years and administration of non-cephalosporin prophylactic or additional antibiotics before or during surgery. SSIs is an infection that occurs within 30 days after a surgical procedure, affecting the incision or deep tissues at the surgical site.

### Ethics approval and consent statement

All methods in this study were carried out in accordance with relevant guidelines and regulations. The study was approved by the Ethics Committee of The Affiliated Guangdong Second Provincial General Hospital of Jinan University, Guangzhou, China (Approval No. 2025-KY-KZ-144-01). Due to the retrospective nature of the study and the use of a de-identified database, the Ethics Committee of The Affiliated Guangdong Second Provincial General Hospital of Jinan University waived the need of obtaining informed consent.

### Smart reminder system for prophylactic intraoperative antibiotic re-dosing

In this study, AIMS were used to collect and manage perioperative data. AIMS is an integrated hardware-software platform embedded within the hospital's electronic health record system, enabling automated and standardized documentation of the entire perioperative process, including preoperative assessment, intraoperative management, and postoperative care. The system also facilitates data retrieval for billing, clinical decision-making, quality improvement, and research purposes (8, 10, 11). In August 2024, we have implemented a smart reminder system for prophylactic intraoperative antibiotic re-dosing in the AIMS. The voice prompt alerts, “Surgical duration has reached 2.5 h and is approaching 3 h. Please consider re-dosing prophylactic antibiotics to prevent infection.” The first reminder is triggered 2.5 h after the surgery begins, with subsequent reminders every 3 h (Supplementary Figure S1). In addition, two inquiry items were added to the preoperative application form in the AIMS: (1) the estimated duration of surgery and (2) whether preparation for intraoperative antibiotic re-dosing had been made. Furthermore, we strengthened the evaluation of medical orders by improving the timing review of perioperative antimicrobial use, using the time recorded in the AIMS as the reference standard. Both Class I and Class II surgical wounds were incorporated into the routine review of perioperative antimicrobial prescriptions.

### Data collection

The data from the Blue Dragon Hospital Infection Real-time Monitoring and Management System (Hunan Blue Dragon Network Technology Co., Ltd., version 6.0) and the Hospital Intelligent Infection Management System (Shanghai Lilian Information Technology Co., Ltd.) are used to collect hospital infection cases. These cases are reviewed and tracked by dedicated infection control specialists in the hospital. In the AIMS (Neusoft Corporation Co., Ltd. V6.0), the following data were collected: surgical duration, preoperative prophylactic medication administration time, time of surgery completion, BMI, BSA, intraoperative additional prophylactic medication administration time, and the names of prophylactic medications. Basic patient information, including gender, age, medical history (hypertension, diabetes, coronary artery disease, hepatitis, tuberculosis, drug or food allergies, surgical history, transfusion history), hospitalization costs, and length of hospitalization, were retrieved from the electronic medical record system (Beijing Jiahe Meikang Information Technology Co., Ltd. V6.0). Surgical time is defined as the moment when surgical instruments first enter the body cavity through a natural orifice to access the surgical site, without any skin or mucosal incisions. The exclusion criteria specify that the start time of surgery must not be substituted by the time of entering the operating room, the initiation of anesthesia, or the date of the surgery. The surgery end time is defined as the point at which the surgical instruments exit the body cavity following the completion of the surgical procedure. According to the exclusion criteria, this must not be replaced by the time of leaving the operating room or the anesthesia recovery time.

### Statistical analyses

All statistical analyses were performed using IBM SPSS statistical software (version 29.0). Categorical variables were compared using the chi-square test. Counted data were described by the number of cases (percentage), and the Kolmogorov-Smirnov test was used to verify the normality of the data. For continuous variables that followed a normal distribution, the mean ± standard deviation (SD) was used to describe them, and the differences between the groups were assessed using Student's *t*-test. For continuous variables that did not follow a normal distribution, data were described using the median and interquartile range (IQR, P25, P75), and group comparisons were performed using non-parametric tests, specifically the Mann-Whitney U test. All statistical analyses were evaluated at the statistical significance level of *p* < 0.05 (two-sided).

## Results

### Demographic information

The rates of prophylactic antibiotic administration before surgery significantly increased after the intervention (*p* = 0.005). The timing of prophylactic antibiotic administration before surgery was shorter after the intervention, with a median of 0.8 h (IQR: 0.6–1.0), compared to the before intervention period (*p* < 0.001). The rates of intraoperative additional antibiotic administration also increased significantly (*p* = 0.003). Furthermore, the rate of SSIs decreased significantly after the intervention (*p* = 0.038). The demographic information before and after the intervention is presented in Table 1.

**Table 1:** Baseline characteristics of patients before and after the intervention.

**Characteristics**	**Before intervention (*n =* 1,145)**	**After intervention (*n =* 1,059)**	***p-*value**
Sex, male	608 (53.10)	586 (55.34)	0.293
Age	57 (46–66)	56 (44–66)	0.169
BMI	23.50 (21.16–26.12)	23.88 (21.19–26.45)	0.121
BSA	1.74 (1.61–1.87)	1.76 (1.63–1.90)	0.086
**Pre-existing comorbidities**			
Hypertension	188 (16.42)	189 (17.85)	0.374
Coronary heart disease	20 (1.75)	23 (2.17)	0.471
Diabetes mellitus	64 (5.59)	82 (7.74)	0.042
Drug/food allergy history	40 (3.49)	33 (3.12)	0.621
Surgical history	375 (32.75)	371 (35.03)	0.258
Hepatitis	16 (1.40)	21 (1.98)	0.285
Tuberculosis	10 (0.87)	6 (0.57)	0.397
Blood transfusion history	24 (2.10)	21 (1.98)	0.851
**Hospital resource utilization**			
Hospitalization cost, CNY	54,106 (41,624–80,382)	55,498 (41,500–82,220)	0.769
Length of stay, days	15 (11–21)	15 (11−21)	0.523
**Surgical characteristics**			
Class I incision (clean)	702 (61.31)	670 (63.27)	0.344
ASA			0.709
I	15 (1.31)	9 (0.85)	
II	799 (69.78)	752 (71.01)	
III	270 (23.58)	254 (23.98)	
IV	49 (4.28)	36 (3.40)	
V	11 (0.96)	7 (0.66)	
VI	1 (0.09)	1 (0.09)	
**Duration of surgery, hours**			
3–6	941 (82.18)	875 (82.63)	0.786
>6	204 (17.82)	184 (17.37)	
**Antibiotic prophylaxis**			
Prophylactic antibiotics administration before surgery	1,055 (92.14)	1,007 (95.09)	0.005
Timing of prophylactic antibiotic before surgery, hours	0.8 (0.6–1.1)	0.8 (0.6–1.0)	<0.001
Intraoperative additional antibiotic	513 (44.80)	541 (51.09)	0.003
Timing of intraoperative additional antibiotic administration, hours	3.0 (2.6–3.5)	3.1 (2.8–3.5)	0.212
**Healthcare-associated infections**			
SSIs	20 (1.75)	8 (0.76)	0.038

Timing refers to the interval between the administration of prophylactic antibiotics and the surgical incision for preoperative use, or the elapsed time from the incision to the administration of an additional intraoperative dose. BMI: Body Mass Index. BSA: Body Surface Area. CNY: Chinese yuan. ASA: American Society of Anesthesiologists Physical Status Classification System. SSIs: Surgical site infections. The *p*-value for ASA classification was calculated using the Chi-square test comparing the distribution across all ASA levels between the two groups. Data are expressed as *n* (%) or median (interquartile range), unless otherwise stated.

### Timing for prophylactic antibiotic administration before surgery and timing for intraoperative additional antibiotic administration

**Figure 2:**
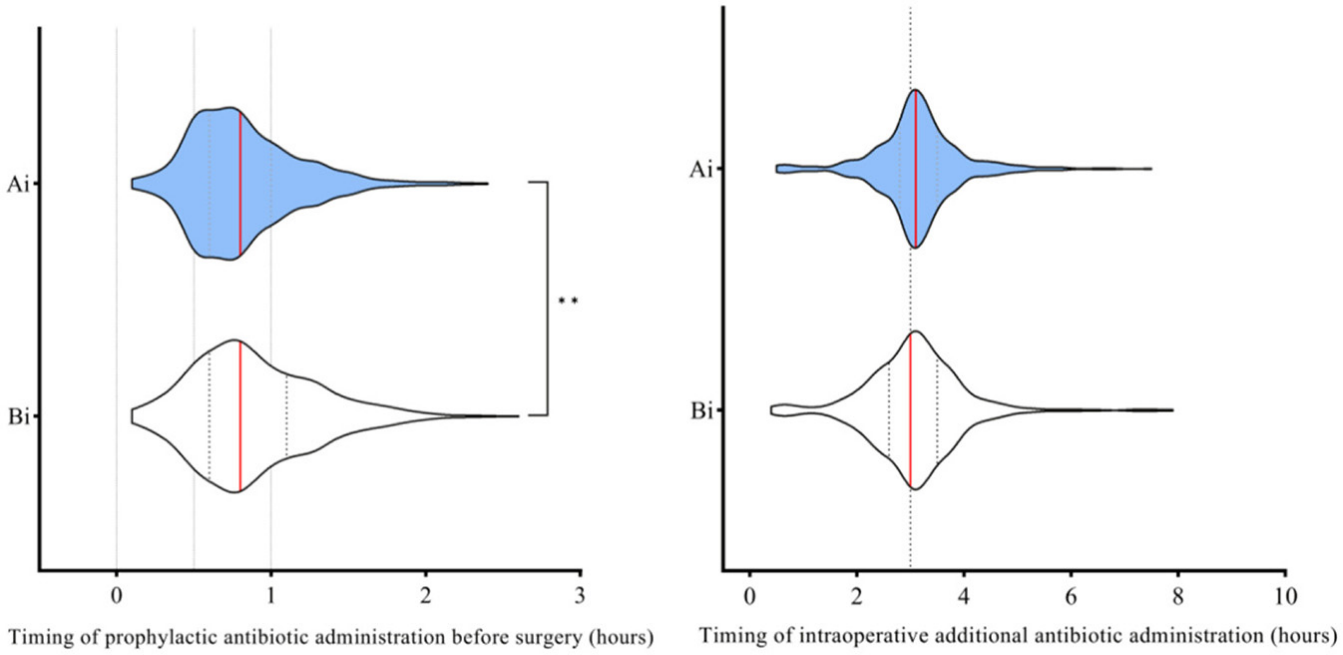
Comparison of timing for prophylactic antibiotic administration before surgery and timing for intraoperative additional antibiotic administration before and after the intervention. Ai, After intervention; Bi, Before intervention. The **red line** represents the median. Timing refers to the interval between the administration of prophylactic antibiotics and the surgical incision for preoperative use, or the elapsed time from the incision to the administration of an additional intraoperative dose. Mann–Whitney U-test was used to evaluate the differences between each item. Symbols for *p*-values: ** <0.001.

After the intervention, the timing for prophylactic antibiotic administration before surgery was shorter (*p* < 0.001), and a higher proportion of patients received antibiotics within a shorter interval compared to before the intervention (Figure 2).

## Discussion

Our study demonstrated that the implementation of a multifaceted intervention, including a smart reminder system, modifications to the preoperative application form, and enhanced review of medical orders, effectively enhanced physicians' compliance with both prophylactic and intraoperative antibiotic administration, which was associated with a significant reduction in SSIs.

Our findings indicated that most demographic, clinical, and surgical baseline characteristics were comparable between the pre-intervention and post-intervention groups (*p* > 0.05), supporting the overall baseline equivalence of the two cohorts. The only statistically significant difference in patient-related factors was the prevalence of diabetes mellitus, which was slightly higher in the post-intervention group (*p* = 0.042). Given that diabetes is a well-recognized risk factor for SSIs, this difference would theoretically predispose the post-intervention group to a higher risk of infection (12). However, patient-related factors such as diabetes mellitus are not easily modifiable, operative time represents a process-related factor that can be influenced through improved perioperative management. Other significant differences in baseline data, such as higher rates of preoperative prophylactic antibiotic administration, shorter prophylactic antibiotic timing intervals, and higher intraoperative additional antibiotic use, were directly related to the intervention and thus represent process improvements rather than confounding variables. The prevalence of diabetes mellitus was higher in the post-intervention group compared with the pre-intervention group (5.59% vs. 7.74%, *p* = 0.042). This higher prevalence would theoretically predispose the post-intervention group to a greater risk of SSIs, yet the observed reduction in infection rates suggests that the improvements were not attributable to baseline differences. Rather, these findings align with the intended mechanism of the smart reminder system and indicate that the intervention likely contributed to the observed decrease in SSI rates.

The prevention of SSIs necessitates a comprehensive strategy that prioritizes the consistent implementation of standardized protocols, including preoperative preparation, adherence to aseptic techniques during surgery, strict hand hygiene, and meticulous postoperative care (7, 13–19). Operative time is an independent risk factor for SSIs and, unlike certain patient-related factors such as diabetes mellitus, it is potentially modifiable (20–22). Prolonged operative time has been shown to increase the risk of developing SSIs. In fact, the majority of studies (87%) have reported a statistically significant association between longer operative duration and the occurrence of SSIs (5). A key factor related to prolonged operative time is the administration of appropriate antibiotic prophylaxis, which includes both preoperative prophylactic antibiotic administration and intraoperative additional dosing (23, 24). The timely administration of an additional dose of prophylactic antibiotics during lengthy surgeries is closely associated with a reduced risk of SSIs (25). Timely intraoperative redosing of antibiotics can reduce the risk of SSIs in patients undergoing prolonged surgery, while also underscoring the critical importance of the initial preoperative prophylactic antibiotic administration (7). Regarding the timing of intraoperative antibiotic redosing, the optimal interval remains unclear. The guidelines from the American Society of Health-System Pharmacists recommend redosing during surgery if the procedure duration exceeds two half-lives of the antibiotic or if there is excessive blood loss (26). Although the benefit of this approach appears reasonable from a pharmacokinetic perspective, current guidelines do not specifically address the duration of surgical procedures or the timing of antibiotic redosing in relation to SSIs within prophylactic antibiotic protocols. In this study, the redosing time was set to approximately 3 h after the start of surgery for procedures lasting longer than about 3–4 h after the initial dose. A smart reminder system was utilized to identify and alert surgeons when intraoperative antibiotic redosing was required for surgeries exceeding 3 h. Since operative duration is not easily modifiable, preoperative estimation of surgery length by the surgeon during scheduling, along with timely reminders and preparation of antibiotics, facilitates prompt intraoperative redosing. Similar to previous studies, our study demonstrates that preoperative prediction of surgical duration, timely reminders for antibiotic preparation, and the implementation of a smart intraoperative redosing reminder system effectively increased the rate of intraoperative antibiotic redosing (27–30).

This study has several limitations that should be acknowledged. First, this study did not analyze important factors among surgical patients, such as maintenance of normothermia, the degree of blood loss, potential breaches in aseptic technique, and the appropriateness of intraoperative nursing care. These factors may also significantly contribute to the occurrence of SSIs, and their omission may have influenced the findings. Second, this is a single-center retrospective study lacking data from randomized controlled trials, which limits the generalizability and strength of the conclusions. Future research efforts should involve large-scale, multicenter randomized controlled trials to address these limitations and validate the effectiveness of the intervention. Third, the follow-up period may not have been sufficient to capture all SSIs, especially those with delayed onset. A longer follow-up duration would help to more accurately assess the true incidence of postoperative infections. Four, we explicitly acknowledge that differences in patient-related factors, such as the higher prevalence of diabetes mellitus in the post-intervention group, may act as potential confounders and could have influenced the results. However, although this baseline difference would theoretically increase the risk of SSIs in the post-intervention group, the observed reduction in SSI incidence indicates that the intervention was effective.

## Conclusion

In summary, the multifaceted intervention, comprising a smart reminder system for prophylactic intraoperative antibiotic re-dosing in the AIMS, preoperative application form modifications, and enhanced medical order review, effectively improved intraoperative antibiotic re-dosing compliance and reduced surgical site infection rates.

## Data Availability

The original contributions presented in the study are included in the article/Supplementary material, further inquiries can be directed to the corresponding author.
