# Supplementary material for: Impact of a multifaceted intervention including a smart reminder system for intraoperative antibiotic re-dosing on surgical site infections in a Chinese tertiary care hospital
Source: Front Public Health. 2025 Oct 10;13:1674811. doi: 10.3389/fpubh.2025.1674811 (PMC12549651; doi:10.3389/fpubh.2025.1674811)

**Supplementary Material**

**Impact of a multifaceted intervention including a smart reminder system for intraoperative antibiotic re-dosing on surgical site infections in a Chinese tertiary care hospital**

*Cuiqiong Fan^1^, Guanwen Lin^1^, Huiwen Zhao^2^, Zhenyao Zhao^3^, Baohong Liu^1^, Tian Wang^1^, Ya Zou^1^, Lushi Huang^4^, Zihuan Li^1*^*

*^1^Department of Infection Prevention and Control, The Affiliated Guangdong Second Provincial General Hospital of Jinan University, Guangzhou, China*

*^2^Department of Preventive Healthcare and Infection Control, Shenzhen People's Hospital (The First Affiliated Hospital, Southern University of Science and Technology; The Second Clinical Medical College, Jinan University), Shenzhen, China*

*^3^Faculty of Humanities and Social Sciences, Hong Kong Metropolitan University, Hong Kong, China*

*^4^Department of Infection Prevention and Control, Guangdong Provincial Social Welfare Service Center (Guangdong Jiangnan Hospital), Guangzhou, China*

**** Correspondence to:***

*Zihuan Li, Department of Infection Prevention and Control, The Affiliated Guangdong Second Provincial General Hospital of Jinan University, Guangzhou, China, lizihuan0102@163.com.*

Number of pages: 2

Number of figures: 1

Figure S1 S2-S2

Figure S1 Smart reminder system for prophylactic intraoperative antibiotic re-dosing


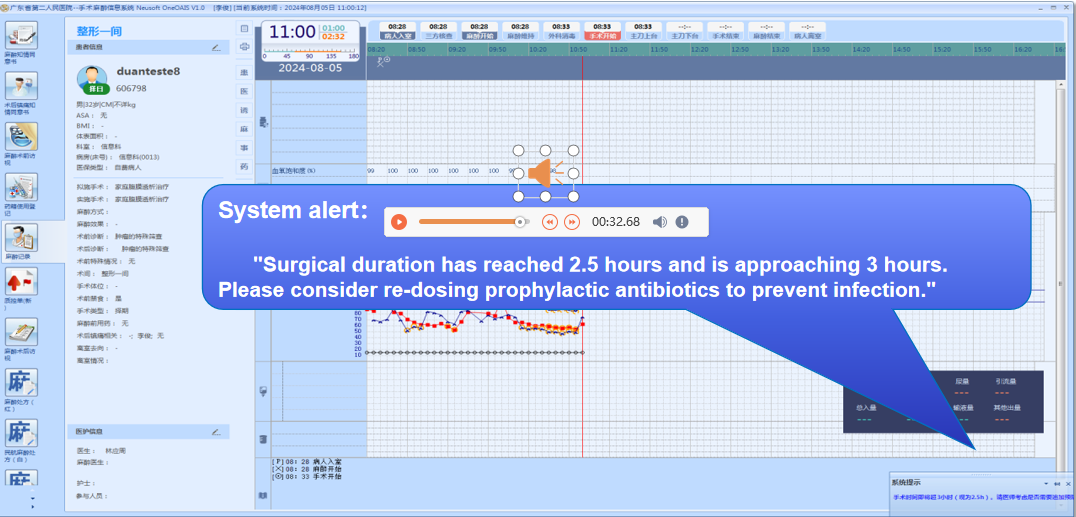

Supplement: Supplementary file 1 [file Data_Sheet_1.doc]
